# Trends in Statin Use in Seniors 1999 to 2013: Time Series Analysis

**DOI:** 10.1371/journal.pone.0158608

**Published:** 2016-07-19

**Authors:** Laura V. Minard, Amber Corkum, Ingrid Sketris, Judith Fisher, Ying Zhang, Ahmed Saleh

**Affiliations:** 1 College of Pharmacy, Dalhousie University, Halifax, Nova Scotia, Canada; 2 Statistical Consulting Unit, Acadia University, Wolfville, Nova Scotia, Canada; 3 Nova Scotia Department of Health and Wellness, Halifax, Nova Scotia, Canada; 4 Faculty of Medicine, Dalhousie University, Halifax, Nova Scotia, Canada; University of British Columbia, CANADA

## Abstract

**Purpose:**

To examine HMG-CoA reductase inhibitor (statin) drug dispensing patterns to Nova Scotia Seniors' Pharmacare program (NSSPP) beneficiaries over a 14-year period in response to: 1) rosuvastatin market entry in 2003, 2) JUPITER trial publication in 2008, and 3) generic atorvastatin availability in 2010.

**Methods:**

All NSSPP beneficiaries who redeemed at least one prescription for a statin from April 1, 1999 to March 31, 2013 were included. Aggregated, anonymous monthly prescription counts were extracted by the Nova Scotia Department of Health and Wellness (Nova Scotia, Canada) and changes in dispensing patterns of statins were measured. Data were analyzed using descriptive analyses and interrupted time series methods.

**Results:**

The percentage of NSSPP beneficiaries dispensed any statin increased from 5.3% in April 1999 to 20.7% in March 2013. In 1999, most NSSPP beneficiaries were dispensed either simvastatin (29.5%) or atorvastatin (28.7%). When rosuvastatin was added to the NSSPP Formulary in August 2003, prescriptions dispensed for simvastatin, lovastatin, pravastatin, and fluvastatin declined significantly (slope change, -0.0027; 95% confidence interval (CI), (-0.0046, -0.0009)). This significant decline continued following the publication of JUPITER (level change, -0.1974; 95% CI, (-0.2991, -0.0957)) and the availability of generic atorvastatin (level change, -0.2436; 95% CI, (-0.3314, -0.1558)). Atorvastatin was not significantly affected by any of the three interventions, although it maintained an overall decreasing trend. Only upon the availability of generic atorvastatin did the upward trend in rosuvastatin use decrease significantly (slope change, -0.0010, 95% CI, (-0.0015, -0.0005)).

**Conclusions:**

The type and rate of statins dispensed to NSSPP beneficiaries changed from 1999 to 2013 in response to the availability of new agents and publication of the JUPITER trial. The overall proportion of NSSPP beneficiaries dispensed a statin increased approximately 4-fold during the study period. In 2013, rosuvastatin was the most commonly dispensed statin (44.1%) followed by atorvastatin (39.1%).

## Introduction

The use of HMG-CoA reductase inhibitor (statin) drugs in the population is increasing with cumulative global sales estimated to approach $1 trillion by 2020 [1]. In Canada, statins are one of the fastest growing drug classes with expenditures increasing from $0.5 billion in 1998 to $1.9 billion (Canadian) in 2007 [2]. There are currently six different statins on the Canadian market; all are available as generic formulations. Many factors affect prescribers' decisions whether to prescribe statins and which statin to prescribe, including published literature related to effectiveness and safety of the drug, clinical practice guidelines, patient’s clinical characteristics, drug insurance benefit coverage, the patient’s ability to pay, patient preferences, and pharmaceutical industry marketing [1, 3–5].

Publication of randomized controlled trials (RCTs) involving statins has been associated not only with increases in the rate of statin use, but also with shifts in the market share of the statin featured in individual trials [6,7]. JUPITER (Justification for the Use of statins in primary Prevention: an Intervention Trial Evaluating Rosuvastatin) was an RCT that examined the effect of rosuvastatin on the occurrence of the combined end point of myocardial infarction (MI), stroke, arterial revascularization, hospitalization for unstable angina, or death from cardiovascular causes among apparently healthy men and women without hyperlipidemia, but with elevated high-sensitivity C-reactive protein levels [8,9]. At the time of JUPITER publication, rosuvastatin was available only as brand name Crestor®.

We examine the potential influence of three specific events on trends in statin utilization in Nova Scotia, Canada. First, Health Canada issued AstraZeneca a notice of compliance to market Crestor® (rosuvastatin) in February 2003. Second, the results of JUPITER were published in November 2008. Third, generic formulations of atorvastatin became available in 2010 upon expiration of the patent for Lipitor®. The purpose of this study was to describe the changes in community pharmacy dispensing of statins to beneficiaries of the Nova Scotia Seniors’ Pharmacare Program (NSSPP) over a 14-year period in response to these three events.

## Methods

### Study population and data sources

Nova Scotia is a Canadian province with a population of 921727; almost 17% are individuals aged 65 or older [10]. The NSSPP is a publicly funded drug insurance plan that reimburses drugs and medical supplies listed in the Nova Scotia Formulary for eligible residents in the province [11]. The beneficiaries of this program are Nova Scotia residents 65 years of age or older who enrolled in the program by paying the required insurance premium and co-payments. The NSSPP does not provide coverage for seniors who have private drug insurance, benefit coverage with Veterans Affairs Canada, or First Nations and Inuit Health. Approximately 85% of seniors in Nova Scotia are eligible beneficiaries under the NSSPP (data on file, Population Health Research Unit, Dalhousie University).

All NSSPP beneficiaries who redeemed at least one statin prescription between April 1, 1999 and March 31, 2013 were included in this study. Table 1 presents details regarding statins that were available to NSSPP beneficiaries during the study period. Throughout the study period, seven statins were available in Canada: atorvastatin, lovastatin, pravastatin, simvastatin, rosuvastatin, fluvastatin and cerivastatin. Cerivastatin was discontinued early in the study period (2001). For some analyses, the lower potency statins (simvastatin, lovastatin, pravastatin and fluvastatin) were combined to form a group of 'other statins' due to declining use of these statins. As of March 31, 2013 all six currently available statins were benefits on the Nova Scotia Formulary without restriction criteria. Nova Scotia has a province wide program, Cardiovascular Health Nova Scotia, which provides guidance, but does not recommend a specific statin (http://novascotia.ca/dhw/cvhns/).

**Table 1:** Statins, manufacturers and dates that brand and generic formulations were first listed on the Nova Scotia Formulary.

Statin^a^	Date listed on Nova Scotia Formulary	
	Brand	Generic
Atorvastatin	October 1, 1997	July 28, 2010
Rosuvastatin	August 15, 2003	April 9, 2012
Simvastatin	December 1991	March 1, 2003
Pravastatin	March 1991	June 1, 2001
Lovastatin	July 1989	1997^**b**^
Fluvastatin	September 1994	March 11, 2013

^a^Cerivastatin: Notice of Compliance February 1998; delisted August 2001; discontinued October 2001

^b^Exact date not located.

Aggregated, anonymous monthly prescription counts were extracted by the Nova Scotia Department of Health and Wellness, and changes in dispensing patterns of statins were measured using descriptive analyses and interrupted time series methods. Ethics approval was granted by the Dalhousie University Research Ethics Board (REB # 2011–2474). All collected and analyzed data was aggregated and anonymous; therefore, informed consent was not obtained.

The percentage of NSSPP beneficiaries dispensed any statin was calculated by dividing the number of beneficiaries who were dispensed a prescription for any statin in a given month by the total number of beneficiaries registered under the program in the corresponding year. To determine the percentage of NSSPP beneficiaries who were dispensed a specific statin, the number of beneficiaries dispensed the specific statin in a given month was divided by the total number of beneficiaries dispensed any statin in the same month.

### Time series analysis

Time series regression analysis or segmented regression for interrupted time series data was performed. The parameters of interest for each segment were the level and/or the trend in the percentage of beneficiaries dispensed 'other statins', atorvastatin or rosuvastatin [12].

There were level and trend changes in all three time series in July 2001, which was not a time point of interest in our study. We therefore began the time series analysis in August 2001. The pre-intervention period was August 2001-July 2003 for 'other statins' (24 time points), May 2006-October 2008 for atorvastatin (30 time points) and August 2004-October 2008 for rosuvastatin (51 time points). The impact of three specific events on the type and rate of statin dispensed to NSSPP beneficiaries was measured using time series regression as described in the next section: 1) August 2003, rosuvastatin entered onto the market, 2) November 2008, JUPITER trial was published, and 3) August 2010, generic atorvastatin became available to NSSPP beneficiaries. On March 11, 2010, a Supplement to a New Drug Submission for Crestor® was approved for a new indication advising its use to reduce the risk of non-fatal MI or stroke, or coronary revascularization, in patients without documented cardiovascular events but with at least two risk factors for cardiovascular disease.

There were 24 time points from the start of the data set in August 2001 until rosuvastatin became available to NSSPP beneficiaries (August 2003), 63 time points between when rosuvastatin became available and publication of the JUPITER trial (November 2008), 21 time points between publication of the JUPITER trial and the availability of generic atorvastatin (August 2010), and 32 time points from the availability of generic atorvastatin to the end of the study period (March 2013).

To determine other sources of change in the clinical practice environment that may have influenced statin prescribing, a literature review of clinical practice guidelines and major clinical trials published during the study period that may have affected dispensing patterns are illustrated in S1 and S2 Tables. Randomized controlled trials of atorvastatin, fluvastatin, lovastatin, pravastatin, rosuvastatin, simvastatin, or cerivastatin, of at least a 12-month duration, were included, and were defined as primary prevention if the majority (>50%) of the population had no history of coronary heart disease. These studies compared a statin with placebo, standard therapy, or no treatment and reported on any of the following clinically important cardiovascular outcomes: all-cause mortality, cardiovascular mortality, fatal MI, nonfatal MI, and major coronary events.

### Statistical modelling

We performed time series regression for three interrupted time series: the percentage of NSSPP beneficiaries per month prescribed 1) ‘other statins’; 2) atorvastatin; and 3) rosuvastatin. All data were examined graphically to observe the overall trends and to identify seasonal patterns and potential outliers. We implemented a two-step parametric approach to assess the impact of the interventions. First, the best general linear model (GLM) for the percentage of beneficiaries in each statin grouping was fitted. A residuals diagnostic check was conducted to examine preliminary model adequacy and possible dependency structure in the data. If the dependence was significant, in the second step, the intervention effects were analyzed by a time series regression model on an appropriate scale with serial autocorrelations and the long-term trend over time was adjusted. A time series regression model can be considered as a regression model with autocorrelated dependent errors. Such errors are usually modeled by an integrated autoregressive moving average model (ARIMA). The regression and ARIMA models may be selected with the information criteria (*Akaike* information criterion and/or the *Bayesian information criterion)* and various residual analysis tools (e.g. Ljung-Box test for dependence, Dickey-Fuller test for stationarity, and autocorrelation/partial autocorrelation function plot for dependence structure).

There are large variations in the percentage of beneficiaries dispensed rosuvastatin prior to August 2004, and in the percentage of beneficiaries dispensed atorvastatin prior to May 2006. The observed large variation may have resulted from the “rosuvastatin entry effect”. As a new drug, its dispensing pattern appears unstable at the entry time period, which may correspondingly cause the nonlinear and unstable dispensing pattern in atorvastatin. In order to carry out adequate time series regression analysis on the percentage of beneficiaries dispensed rosuvastatin, all time series points prior to August 2004 had to be removed, and on the percentage of beneficiaries dispensed atorvastatin, all time series points prior to May 2006 had to be removed. Removing sections of the time series is common in time series intervention analysis [12]. The model diagnostic checks indicated the selected models were reasonably adequate to fit the cleaned data. Data analysis was performed using R statistical software 2.12.0. (R Foundation for Statistical Computing).

## Results

**Fig 1:**
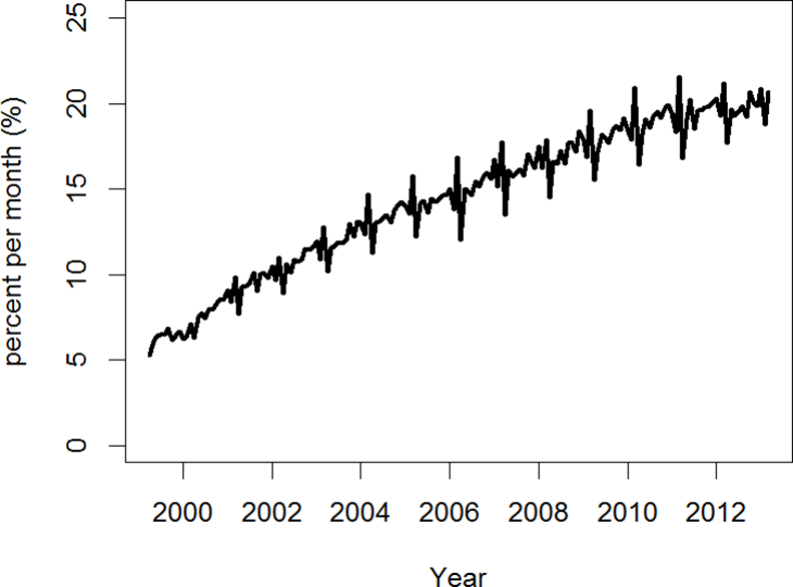
Percentage of Nova Scotia Seniors' Pharmacare Program (NSSPP) beneficiaries dispensed any statin over the 14-year period from April 1999 to March 2013. The number of NSSPP beneficiaries per month who were dispensed a prescription for any statin in a given month was divided by the total number of NSSPP beneficiaries registered under the program in the corresponding year.

**Fig 2:**
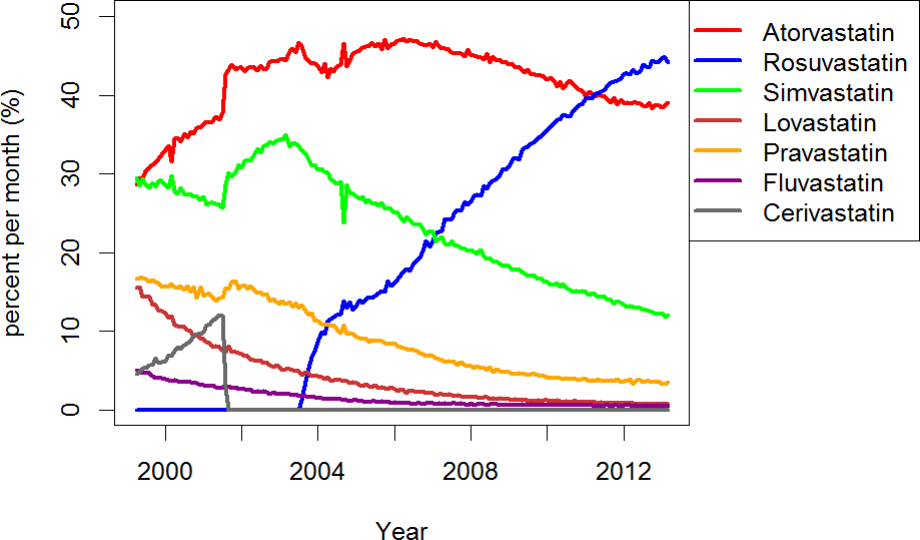
Statin market share of prescriptions dispensed to Nova Scotia Seniors' Pharmacare Program (NSSPP) beneficiaries in each month between April 1999 and March 2013. The number of NSSPP beneficiaries dispensed each statin in a given month was divided by the total number of beneficiaries dispensed any statin in the same month.

The percentage of NSSPP beneficiaries dispensed any statin per month from community pharmacies increased from 5.3% in 1999 to 20.7% in 2013 (Fig 1). The percentage of NSSPP beneficiaries dispensed each type of statin per month during the 14-year period is shown in Fig 2. Statin market share changed over the study period. In 1999, most NSSPP beneficiaries were dispensed simvastatin (29.5%) or atorvastatin (28.7%); however, the most common type of statin dispensed in March 2013 was rosuvastatin (44.1%).

**Fig 3:**
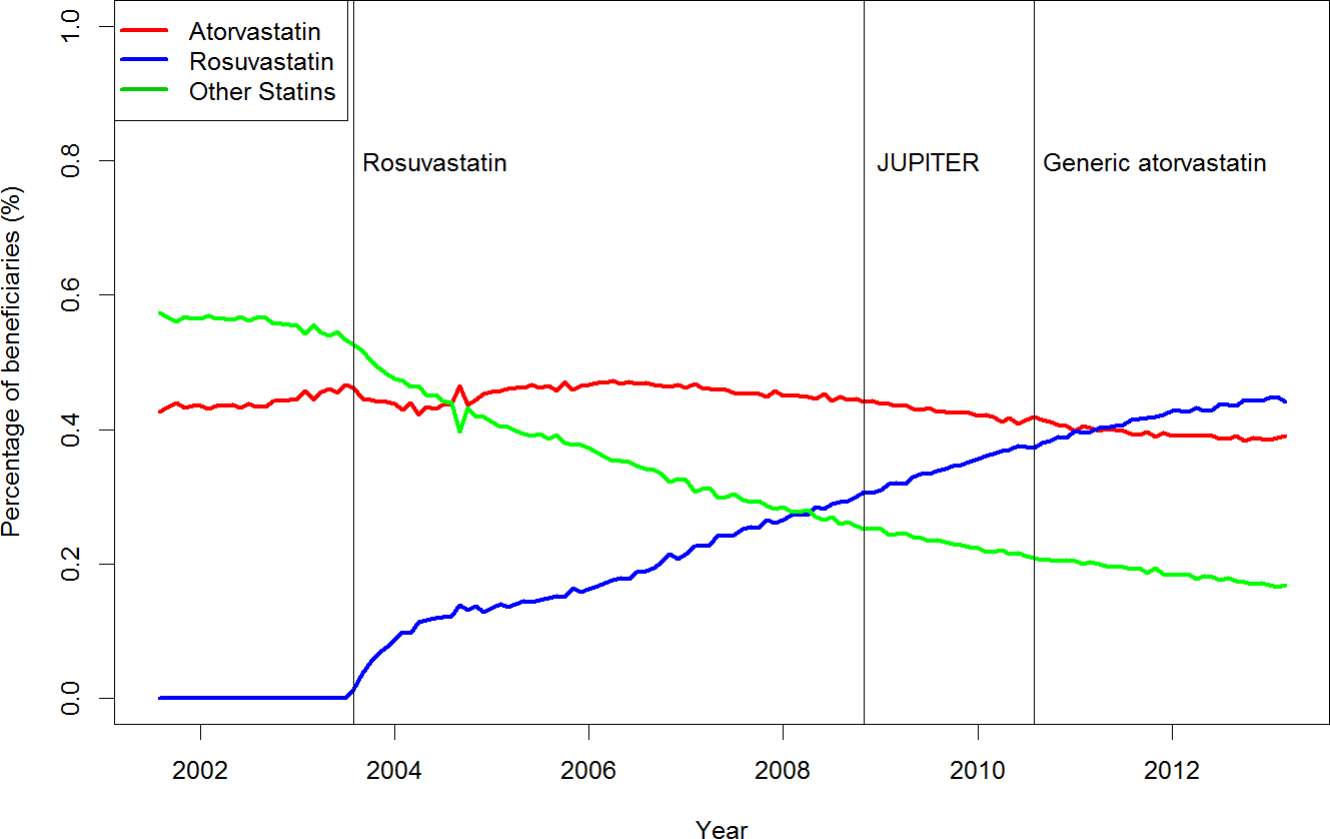
The type and rate of statin dispensed to Nova Scotia Seniors' Pharmacare Program beneficiaries from August 2001 to March 2013 was analyzed using time series analysis. The total number of beneficiaries dispensed a statin was used as the denominator in calculations. 'Other statins' includes simvastatin, fluvastatin, pravastatin and lovastatin.

**Fig 4:**
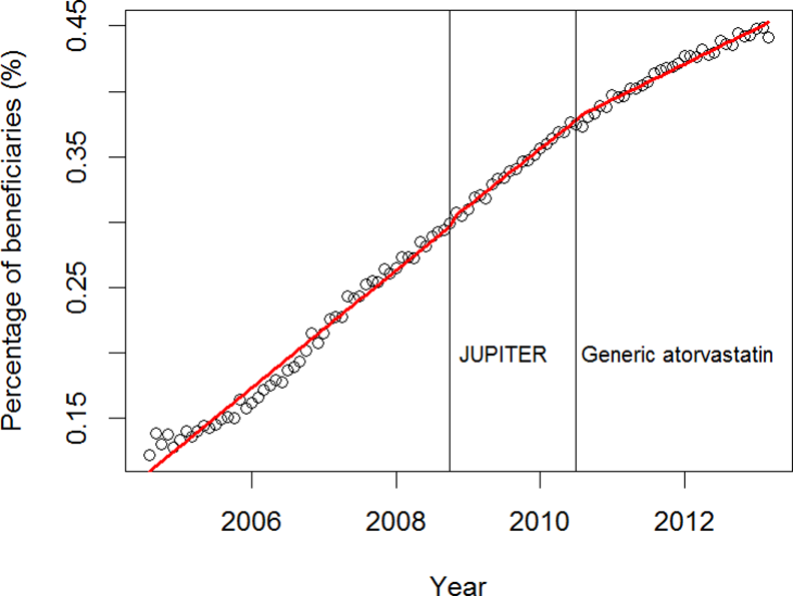
The rate of increasing trend in the percentage of Nova Scotia Seniors' Pharmacare Program beneficiaries dispensed rosuvastatin decreased significantly upon the availability of generic atorvastatin in August 2010. Time series analysis and a two-stage modelling approach were used. The total number of beneficiaries dispensed a statin were used as the denominator in percentage calculations. Individual time points are represented by open circles. Fitted values are illustrated using a red line.

The type and rate of statin dispensed during the study period was analyzed using time series analysis. Fig 3 presents a simultaneous plot to show the trends of 'other statins', atorvastatin and rosuvastatin. Overall, dispensations for ‘other statins’ and atorvastatin decreased while dispensations for rosuvastatin increased. However, the changes in the trend of the individual time series cannot be easily observed due to the large variation and scale in this plot. We thus conducted time series analysis for each of the three statin groupings separately (Fig 4, S1–S3 Figs). It is important to note that for each time series analysis, we estimated the trend and/or level change due to each intervention and compared it to the decreasing/increasing baseline trend and/or overall mean level rather than to that from other interventions (e.g. the slope of the line after the availability of generic atorvastatin was compared to the slope of the baseline, rather than the slope of the line after the publication of JUPITER).

Controlling for an overall decreasing trend, when rosuvastatin was added to the Nova Scotia Formulary in August 2003, the percentage of beneficiaries dispensed 'other statins' (simvastatin, lovastatin, pravastatin, fluvastatin) significantly decreased further (slope change, -0.0027; 95% confidence interval (CI), (-0.0046, -0.0009)) (S1 Fig). There was also an overall decreasing trend in the percentage of beneficiaries dispensed atorvastatin (S2 Fig).

Controlling for baseline trends, following the publication of JUPITER in November 2008, the percentage of beneficiaries dispensed 'other statins' significantly declined (level change -0.1974; 95% CI, (-0.2991, -0.0957)) (S1 Fig). The percentage of beneficiaries dispensed atorvastatin was not significantly affected by JUPITER; however, the overall decreasing trend continued (S2 Fig). The percentage of beneficiaries dispensed rosuvastatin did not significantly change as a result of JUPITER, however the overall increasing trend continued (Fig 4 and S3 Fig).

Controlling for baseline trends, when generic formulations of atorvastatin became available to NSSPP beneficiaries in August 2010, ‘other statins’ showed a significant level change (-0.2436; 95% CI, (-0.3314, -0.1558)) (Fig 3 and S1 Fig). The percentage of beneficiaries receiving atorvastatin was not significantly affected by the availability of generic atorvastatin; however, the overall decreasing trend continued (Fig 3 and S2 Fig). Rosuvastatin showed a significant trend slope change (-0.0010, 95% CI (-0.0015, -0.0005)), although it maintained an overall increasing trend and slope of 0.0034 (95% CI (0.0029, 0.0039)) (Figs 3 and 4, and S3 Fig).

## Discussion

The type and rate of statins dispensed over the 14-year period from 1999 to 2013 changed in response to the availability of new agents and the publication of JUPITER. In particular, the overall proportion of NSSPP beneficiaries dispensed a statin increased approximately 4-fold during the study period. In addition, the distribution of dispensed statins changed from favoring simvastatin and atorvastatin in 1999 to favor rosuvastatin and atorvastatin in 2013. Three specific events were associated with statistically significant changes in the type and rate of statin dispensed during the study period: rosuvastatin entry onto the market in 2003, publication of JUPITER [9] in 2008, and the availability of generic atorvastatin to NSSPP beneficiaries in 2010.

### Statin utilization among Nova Scotia seniors from 1999 to 2013

Our study showed an increase of approximately 4-fold in monthly dispensations of statins for NSSPP beneficiaries from 5.3% in April 1999 to 20.7% in March 2013. This increase is consistent with the findings of other studies [13–17]. Reasons for the increase in statin dispensations include a larger proportion of the population being treated, treating patients more intensively according to new guidelines or treating to lower target, or patients increasing medication adherence.

In a European study including nine countries, statin utilization (as measured by defined daily doses (DDDs)/1000 inhabitants/day) varied widely across countries [13]. All countries experienced an increase in statin utilization from 2000 to 2003 with the largest increase occurring in Ireland (3.74-fold), and the smallest increase occurring in France (1.56-fold) [13]. Walley et al found that statin utilization in Finland increased 2.11-fold; however, in another study of longer duration, the annual number of ongoing statin users increased 4.6-fold between 1999 and 2008 [13,16]. Although Walley et al found only a 1.58-fold increase in statin utilization in Belgium from 2000 to 2003, Fraeyman et al, also measuring DDDs/1000 inhabitants/day, found a 20-fold increase from 1997–2009 [13,15]. In Denmark, statin treatment prevalence increased 27-fold from 1996 to 2009 [14]. In the US, Charlesworth et al, using the National Health and Nutrition Examination Survey (NHANES), found that the proportion of adults aged 65 or older taking a statin increased approximately 23-fold from 1988 (2%) to 2010 (45.7%) [17]. The reasons for the variations in the rates of increase of statin utilization between different jurisdictions are unclear. The larger increase in the US may be related to marketing by the pharmaceutical industry including direct-to-consumer advertising (DTCA). Direct-to-consumer advertising in Canada occurs through reminder ads and statins were one of the top classes marketed during part of our study period [18]. While the European Commission provides guidance on patient-specific information, DTCA is not allowed in the same manner as in the US. However, in Europe, statin utilization also varied.

### Effect of publication of JUPITER on statin utilization

Our study showed that publication of JUPITER did not lead to a significant change in the rate of rosuvastatin or atorvastatin dispensing, but that the percentage of beneficiaries dispensed 'other statins' declined. In another Canadian study, Teng et al examined the effect of the publication of JUPITER on incident statin prescribing for primary prevention from 2003 to 2011 and determined that it did not impact the dispensation of rosuvastatin or all statins during this period; however, the market share of the incident rate of dispensations of rosuvastatin increased from 9% to 65% [19].

In our study, the percentage of beneficiaries per month dispensed a statin following publication of JUPITER increased over time from 17.2% in November 2008 to 21.7% in March 2013. Although statin dispensations increased after JUPITER the increase was not as high as would be expected if prescribers were to use the JUPITER eligibility criteria to treat all patients, as found by other researchers [20,21]. For example, Galper et al estimated that the percentage of the population taking statins if treated according to the eligibility criteria and recommendations from ATP III [22] compared to JUPITER [9] was 31.0% and 41.9% for men aged 45–75, and 17.5% and 53.1% for women aged 55–75 [23].

Several factors may explain why we did not see an increase in the rate of rosuvastatin dispensations following the JUPITER trial, including perceived limitations of the trial, concern about the risk-benefit of statins prescribed to patients for primary prevention, and the limited clinical utility of hs-CRP levels [9,24]. Of note, the trial was terminated early because the incidence of cardiac events in the rosuvastatin group was significantly reduced [9,25,26], and was followed by substantial media attention [25]. The study findings remain controversial and their interpretation has been the subject of considerable debate [25, 27–38].

### Effect of expiration of Lipitor® patent

Our study did not find a significant increase in atorvastatin dispensations upon the availability of generic atorvastatin. However, we were not able to study incident prescriptions and prescribers may have wanted to leave patients on existing therapies. In Korea, unlike Nova Scotia, once generic atorvastatin entered the market place, there was a 240% increase in the number of patients prescribed generic atorvastatin from July 2008 (date of patent expiry) to June 2010, with greater increases in the newly treated group [39]. Physicians in some jurisdictions may have limited trust in generic medications [40]; however, there is no evidence that this is the case in Nova Scotia. The NSSPP generally follows Health Canada recommendations regarding bioequivalence of generic brands, and evidence from meta-analysis suggests that comparable results are found between generic and brand-name statin drugs [41].

Other factors may have also contributed to the limited increase in atorvastatin market share in Nova Scotia following its patent expiry. The NSSPP does not provide financial incentives for physicians and did not have a prior authorization policy related to Lipitor® (atorvastatin) while it remained on patent. Shrank et al noted that Lipitor® prior authorization requirements in some US Medicaid programs were associated with 30.6% lower Lipitor® use [42]. Nova Scotia primary care physicians also did not have a computerized decision support system that provided a default option to specific generic statin medications. One US study demonstrated that such a decision support system could increase the prescribing of generic statins [43]. In addition, prescribers may have been concerned with atorvastatin liver toxicity; the Prove It trial used high atorvastatin doses (80 mg) and resulted in a higher percentage of patients with elevations in alanine aminotransferase levels compared to standard dose pravastatin [44].

### Effect of availability of higher potency statin products

Our study found that the use of lower potency, 'other statins', decreased over time in Nova Scotia, while these agents increased in some other jurisdictions. A 2005 Dalhousie academic detailing initiative focused on risk reduction in primary and secondary prevention and noted that lovastatin, pravastatin and simvastatin were subject to a maximum allowable cost policy, and compared costs of all statin products on the Nova Scotia market [45]. Physicians are not always aware of price differences in drugs of the same class and do not necessarily choose the least expensive drug [46,47]. Other studies have found that reimbursement policies can influence the uptake of preferred statins [48]. All statins were full benefits on the Nova Scotia Formulary; therefore, the formulary did not influence the prescribing of specific statins. Because Nova Scotia did not have a tiered formulary, where patient copayments varied greatly by type of statin dispensed, physicians and patients may not have been price-sensitive. Conversely, a US study of 12 Fortune 500 firms found that following the expiration of the patent for Zocor®, simvastatin prescriptions increased significantly, especially for those patients with lower incomes [49]. Specifically, simvastatin prescriptions climbed from just under 16% of the initial statin prescribing market share in 2005 (as measured by pharmacy dispensations) to over 40% at the end of 2007 [49]. In Sweden, Lindgren et al noted that in 2008, 80% of patients treated with lipid lowering drugs were treated with simvastatin whereas in Nova Scotia in 2008 this was only 19% [50].

Atorvastatin and rosuvastatin may have been preferred by physicians due to perceived increased efficacy, higher potency, advertising expenditures, provision of samples to physicians and coupons to patients, or other factors. Physicians may have been provided with samples for drugs that remained on patent. While we did not have Canadian data, in the US statins were the most frequently sampled prescription class by volume from 2009 to 2011 and second in 2012 and 2013. Rosuvastatin was the number one sampled individual drug from 2009 to 2011 and atorvastatin the second most sampled drug in 2009 and 2010 [51]. Physicians may also have developed mindlines, habits and rules of thumb for prescribing a specific statin regimen using evidence, their own experience and that of their peers, social context and other factors, and these can be difficult to change by only providing written material (e.g. guidelines) [52,53].

Direct-to-consumer advertising, especially from US television ads, may have increased awareness of cardiovascular disease and increased the overall demand for statins as well as for specific statins, but may have provided consumers with limited information on risks and benefits, and patients need decision support tools which can be used with the health care provider to adapt guidelines to their own circumstances [54]. In Canada, where only reminder advertising is allowed, atorvastatin (Lipitor®) was the fourth highest brand by advertising spending, all media, for 2001 to 2006 at $8.12 million (Canadian) [18].

Prescribers select statins based on many factors including potency (rosuvastatin and atorvastatin are considered high potency), degree of lipid lowering or target lipid level, other concomitant drugs and diseases (e.g. where the type of metabolizing liver enzyme, route of excretion or lipophilicity may play a role), adverse effect profile, strength of clinical trial evidence and the population studied in the trials, and cost. In addition, prescribers may be influenced by pharmaceutical marketing, continuing professional education and academic detailing [55]. The increase in higher potency statins may be due to the prescribers' desire to adhere to newer guidelines with lower LDL targets which were highly promoted. Cardiovascular disease with a focus on statins was the subject of two Dalhousie academic detailing programs in Nova Scotia during the study period [45,56], which may also have influenced prescribing.

### Strengths and limitations

This study has a number of strengths. Importantly, the longitudinal nature allowed us to study statin dispensing patterns over a 14-year period in a stable population. We were able to capture this information for approximately 73% of Nova Scotia residents over the age of 65 [10]. Details of the pharmaceutical reimbursement policy were known during this time period, as well as information regarding educational initiatives provided by Dalhousie Continuing Medical Education Academic Detailing Services. Time series analysis allowed us to study the impact of three events on the type and rate of statin dispensed to NSSPP beneficiaries over time.

A number of limitations need to be considered. First, the analysis was based on drugs that were dispensed, not prescribed or consumed. Second, although the drugs studied were dispensed by community pharmacies, it is unknown whether the type of statin prescribed was influenced by prescriber affiliation (i.e., community versus hospital practice). Third, we did not examine other factors which could have impacted dispensing patterns such as changes in patient cost sharing, pharmaceutical industry marketing, and continuing professional education events other than those sponsored by the Dalhousie College of Pharmacy and Faculty of Medicine. Fourth, the data were not adjusted for age or sex; however, Nova Scotia census data indicates that the sex distribution of the population remained similar over the study period [10]. The proportion of Nova Scotians over age 65 increased from 13.9% in 2001 to 16.6% in 2011 [10]. Finally, we examined aggregated data and were not able to link data to patient health information; therefore, we were unable to determine prescribing for specific age groups of beneficiaries, the reason for prescribing (e.g., patient risk factors, history of cardiovascular disease, lipid profile), the therapeutic regimen (e.g. dose prescribed), therapeutic intent and monitoring, patient adherence to drug therapy, and patient outcomes. We were not able to study the changes in incidence of prescribing of specific statins. Incident prescriptions may have been more sensitive to change. We were also not able to examine statin type by prescriber specialty, which may be an important factor in which statin individual prescribers choose. For example, different specialty societies may recommend specific statins for particular disease conditions.

A common limitation of the statistical models is the presence of some weak dependence between the residuals. Significant level and trend changes were determined via comparison to the pre-intervention periods. Also, the three statin groupings were analysed individually without considering any correlation among them.

### Next steps

The analysis of the type, rate, costs and outcomes of statin prescribing over time by prescriber and type of patient will become increasingly important as the 2013 American College of Cardiology/American Heart Association (ACC/AHA) guidelines on the treatment of cholesterol [57] are implemented. This may result in a larger number of patients being treated, especially patients over 75 for whom the guidelines provide specific recommendations [23,57]. In addition, there is a need for careful communication of benefits, risks and uncertainties for individual patients, especially those receiving statins for primary prevention, those at the end of life, etc [23,58,59].

Further work is needed to examine the reasons for prescribers' decisions to prescribe a statin (e.g. cardiovascular risk factors or history of cardiovascular disease), the type and dose of statin, and differences in statin utilization among different age strata of seniors (e.g. age 65–75, 75–85, etc). Future research should also address the effect of providing prescribers with educational interventions and information technology tools to promote evidence-informed cost-effective statin prescribing.

## Conclusions

The type and rate of statins dispensed to beneficiaries of the Nova Scotia Seniors' Pharmacare Program (NSSPP) were determined over a 14 year period. The percentage of NSSPP beneficiaries dispensed any statin per month increased approximately 4-fold from 5.3% in April 1999 to 20.7% in March 2013. Following market entry of rosuvastatin in August 2003, rosuvastatin gained market share such that by March 2013 44.1% of all statin prescriptions for beneficiaries were for rosuvastatin, while 39% were for atorvastatin (versus 46% in August 2003) and 17% were for 'other statins' (simvastatin, lovastatin, pravastatin and fluvastatin) (versus 53% in August 2003). Publication of JUPITER did not lead to a significant change in the rate of rosuvastatin or atorvastatin dispensing, but led to a decline in the percentage of beneficiaries dispensed 'other statins'. The availability of generic atorvastatin led to a decline in the rate of increase of rosuvastatin utilization and to a further decline in simvastatin, lovastatin, pravastatin and fluvastatin dispensations.

## Supporting Information

S1 Fig The percentage of Nova Scotia Seniors' Pharmacare Program beneficiaries dispensed either simvastatin, fluvastatin, pravastatin or lovastatin declined significantly when rosuvastatin entered the Canadian market (August 2003), after publication of the JUPITER trial (November 2008), and when generic atorvastatin entered the Canadian market (August 2010). Time series analysis and a two-stage modelling approach were used. The total number of beneficiaries dispensed a statin were used as the denominator in percentage calculations. Individual time points are represented by open circles. One outlier is represented by a solid green circle. Fitted values are illustrated using a red line.
(TIFF)

S2 Fig The percentage of Nova Scotia Seniors' Pharmacare Program beneficiaries dispensed atorvastatin did not change significantly when rosuvastatin entered the Canadian market (August 2003), upon publication of the JUPITER trial (November 2008), or when generic atorvastatin entered the Canadian market (August 2010). Time series analysis and a two-stage modelling approach were used. The total number of beneficiaries dispensed a statin were used as the denominator in percentage calculations. Individual time points are represented by open circles.
(TIFF)

S3 Fig The rate of increasing trend in the percentage of Nova Scotia Seniors' Pharmacare Program beneficiaries dispensed rosuvastatin decreased significantly upon the availability of generic atorvastatin in August 2010. Time series analysis and a two-stage modelling approach were used. The total number of beneficiaries dispensed a statin were used as the denominator in percentage calculations. Individual time points are represented by open circles.
(TIFF)

S1 Table Key statin clinical trials published during the study period. (DOCX)

S2 Table Key statin guidelines published during the study period. (DOCX)
