# Supplementary figures and images for: Trends in Statin Use in Seniors 1999 to 2013: Time Series Analysis
Source: PLoS One. 2016 Jul 19;11(7):e0158608. doi: 10.1371/journal.pone.0158608 (PMC4951112; doi:10.1371/journal.pone.0158608)

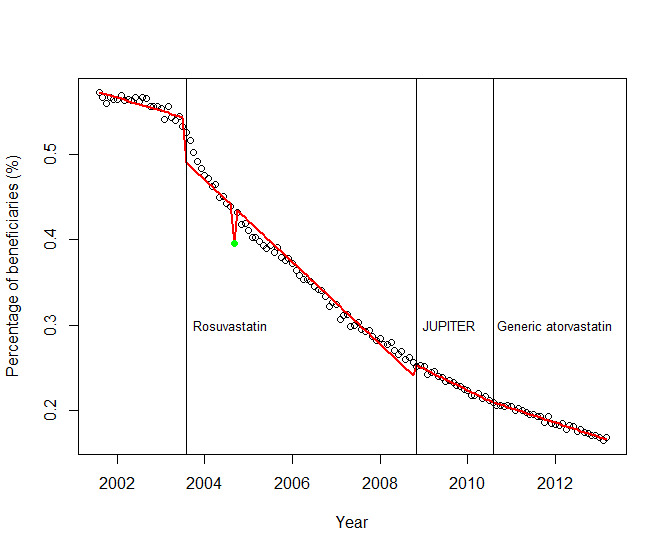

Supplement: S1 Fig — Time series analysis and a two-stage modelling approach were used. The total number of beneficiaries dispensed a statin were used as the denominator in percentage calculations. Individual time points are represented by open circles. One outlier is represented by a solid green circle. Fitted values are illustrated using a red line. (TIFF) [file pone.0158608.s001.tiff]

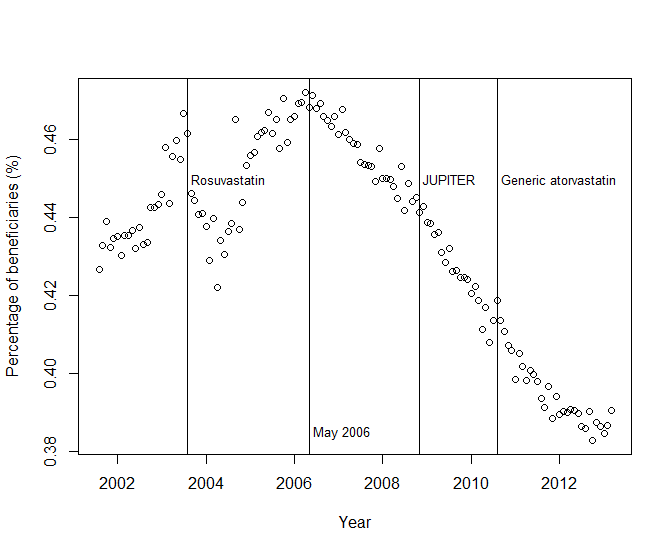

Supplement: S2 Fig — Time series analysis and a two-stage modelling approach were used. The total number of beneficiaries dispensed a statin were used as the denominator in percentage calculations. Individual time points are represented by open circles. (TIFF) [file pone.0158608.s002.tiff]

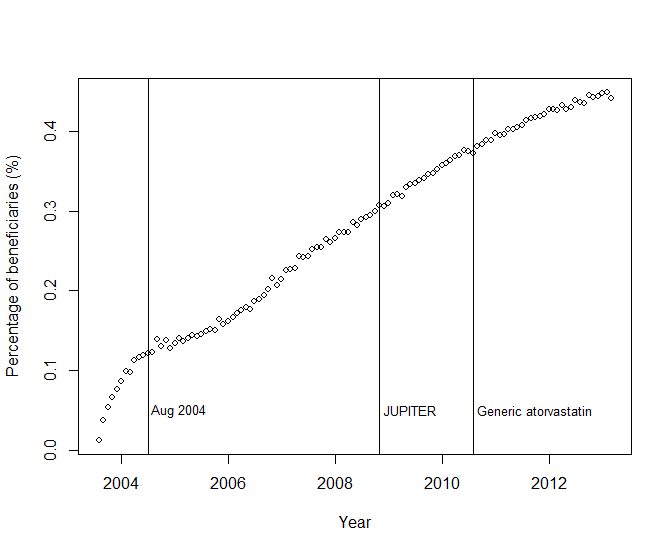

Supplement: S3 Fig — Time series analysis and a two-stage modelling approach were used. The total number of beneficiaries dispensed a statin were used as the denominator in percentage calculations. Individual time points are represented by open circles. (TIFF) [file pone.0158608.s003.tiff]
