# Supplementary material for: Trends in Statin Use in Seniors 1999 to 2013: Time Series Analysis
Source: PLoS One. 2016 Jul 19;11(7):e0158608. doi: 10.1371/journal.pone.0158608 (PMC4951112; doi:10.1371/journal.pone.0158608)
Supplement: S1 Table — (DOCX) [file pone.0158608.s004.docx]

**S1 Table.** **Key statin clinical trials and guidelines published during the study period^a^.**

| Clinical Trials (DD/MM/YYYY) | |
| --- | --- |
| Atorvastatin | 23/09/2003-Mohler III |
|  | 08/04/2004-PROVE IT |
|  | 21/08/2004-CARDS |
|  | 04/04/2001-MIRACL |
|  | 03/03/2004-REVERSAL |
|  | 07/04/2005-TNT |
|  | 28/05/2005-ASCOT |
|  | 21/07/2005-4D |
|  | 16/11/2005-IDEAL |
|  | 29/07/2006-ASPEN |
|  | 10/08/2006-SPARCL |
|  | 13/02/2007-SAGE |
| Rosuvastatin | 06/10/2004-GISSI-HF |
|  | 29/11/2007-CORONA |
|  | 28/03/2007-METEOR |
|  | 20/11/2008-JUPITER |
|  | 02/04/2009-AURORA |
| Cerivastatin | 01/02/2004-Laufs |
| Fluvastatin | 03/04/2001-BCAPS |
|  | 14/06/2003-ALERT |
|  | 01/02/2005-HYRIM |
| Lovastatin | 20/10/1999-CLAPT |
| Pravastatin | 12/05/2000-KLIS |
|  | 15/05/2001-LIPID (subgroup) |
|  | 20/02/2002-FAST |
|  | 18/12/2002-ALLHAT-LLT |
|  | 23/11/2002-PROSPER |
|  | 08/04/2004-PROVE IT TIMI 22 |
|  | 01/07/2004-PACT |
|  | 02/11/2004-PREVEND IT |
|  | 01/12/2004-PHYLLIS |
|  | 30/09/2006-MEGA |
| Simvastatin | 06/07/2002-MRC/BHF |
|  | 14/06/2003-HPS |
|  | 01/03/2005-Hong |
|  | 01/11/2007-SEARCH |
|  | 25/09/2008-SEAS |

^a^References available from authors upon request.
