# Supplementary material for: Trends in Statin Use in Seniors 1999 to 2013: Time Series Analysis
Source: PLoS One. 2016 Jul 19;11(7):e0158608. doi: 10.1371/journal.pone.0158608 (PMC4951112; doi:10.1371/journal.pone.0158608)
Supplement: S2 Table — (DOCX) [file pone.0158608.s005.docx]

**S2 Table.** **Key statin guidelines published during the study period^a^.**

| Guidelines (DD/MM/YYYY) | |
| --- | --- |
| Canadian | 16/05/2000 |
|  | 28/10/2003 |
|  | 22/09/2006 |
|  | 25/10/2009 |
|  | 29/11/2012 |
| US | 16/05/2001-NCEP ATP III |
|  | 01/11/2001-AHA/ACC Guidelines |
|  | 13/07/2004-NCEP ATP III Update 2004 |
|  | 01/05/2006-AHA/ACC Guidelines |
| Joint European Societies | 24/09/2003 |
| Joint British Societies | 01/12/2005 |
| NICE | 01/05/2008 |
| Australia | 05/11/2001 |
| New Zealand | 01/12/2003 |

^a^References available from authors upon request.
